# Prevalence of Hyperhomocysteinemia in China: An Updated Meta-Analysis

**DOI:** 10.3390/biology10100959

**Published:** 2021-09-26

**Authors:** Yuan Zeng, Fei-Fei Li, Shu-Qian Yuan, Hao-Kai Tang, Jun-Hua Zhou, Quan-Yuan He, Julien S. Baker, Yan-Hui Dong, Yi-De Yang

**Affiliations:** 1 Key Laboratory of Molecular Epidemiology of Hunan Province, School of Medicine, Hunan Normal University, Changsha 410081, China; yuanZ@hunnu.edu.cn (Y.Z.); shuqianY@hunnu.edu.cn (S.-Q.Y.); tanghk@hunnu.edu.cn (H.-K.T.); zhoujunhua@hunnu.edu.cn (J.-H.Z.); hqyone@hunnu.edu.cn (Q.-Y.H.); 2 Centre for Health and Exercise Science Research, Hong Kong Baptist University, Kowloon Tong, Hong Kong 999077, China; lifeifei@hkbu.edu.hk; 3 Centre for Health and Exercise Science Research, Department of Sport, Physical Education and Health, Hong Kong Baptist University, Kowloon Tong, Hong Kong 999077, China; 4 Institute of Child and Adolescent Health, School of Public Health, Peking University Health Science Center, Beijing 100191, China

**Keywords:** hyperhomocysteinemia, prevalence, China, updated meta-analysis

## Abstract

**Simple Summary:**

Hyperhomocysteinemia has been defined as an elevated serum concentration of homocysteine exceeding 15 μmol/L and has been proven to play an important role in the pathogenesis of cerebrovascular disease. The prevalence of hyperhomocysteinemia in China has been outlined in a previous meta-analysis. Considering the key role of homocysteine in the process of vascular injury, more studies have been conducted to prevent hyperhomocysteinemia by nutritional supplements such as folic acid or other treatments. Additionally, studies have shown that the prevalence of hyperhomocysteinemia increases over time; therefore, it was necessary to provide an update from the previous meta-analysis on homocysteine status in China. This was needed to understand the prevalence, the trend in changes over time, and its determinants. The results highlight that the prevalence of hyperhomocysteinemia is increasing in China, especially among the elderly, men, and residents in the north, inland areas, and rural areas of China.

**Abstract:**

We conducted a meta-analysis to systematically assess the prevalence of hyperhomocysteinemia (HHcy) in China, its change over time, and its determinants. Literature searches were conducted using English databases (PubMed, Embase, and Web of Science) and Chinese databases (CNKI, CBM, VIP, and Wanfang). The time ranges were from Jan 2014 to Mar 2021 in China. We adopted the random effects model to estimate the pooled positive rates of HHcy and corresponding 95% confidence intervals (95% CI). To find the sources of heterogeneity, we performed subgroup analysis and meta-regression. A total of 29 related articles were identified involving 338,660 participants with 128,147 HHcy cases. The estimated prevalence of HHcy in China was 37.2% (95% CI: 32.6–41.8%, I^2^ = 99.8%, *p* for heterogeneity < 0.001). The trend of HHcy prevalence was gradually upward over time, with increases during 2015–2016 (comparison to 2013–2014, *p* < 0.001), but steady between 2015–2016 and 2017–2018. Subgroup analysis showed that the prevalence was higher in the elderly over 55 years old, males, and residents in the north, inland, and rural China (for each comparison, *p* < 0.001). Meta-regression analysis revealed that age and area of study contributed to 42.3% of the heterogeneity between studies. The current meta-analysis provides strong evidence that the prevalence of HHcy is increasing in China, and varies substantially across different ages, genders, and geographic distribution. Accordingly, high-risk population groups should be focused on, and public health policies and strategies should be carried out to prevent and control HHcy in China.

## 1. Introduction

**Figure 1:**
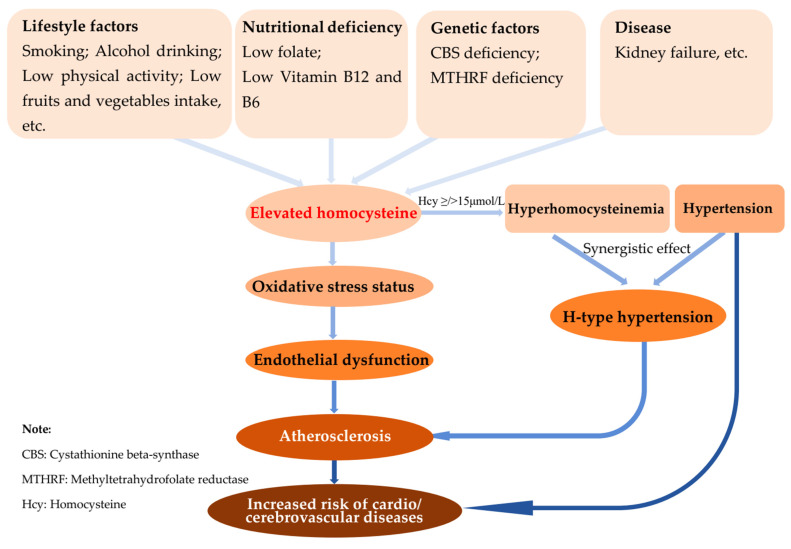
Schematic diagram of the role of homocysteine in the pathogenesis of cardio- and cerebrovascular diseases.

Hyperhomocysteinemia (HHcy) has been defined as elevated serum concentrations of homocysteine (Hcy) exceeding 15 μmol/L [1,2], which have been implicated in contributing an important role in the pathogenesis of cardio- and cerebrovascular disease (Figure 1) [3]. HHcy has been associated with the risk of cardiovascular diseases, such as cerebral infarction and atherosclerosis, especially during the coexistence of HHcy and metabolic syndrome, which highly contribute to cardiovascular diseases [4,5,6]. Numerous studies investigated the prevalence of HHcy and its determinants to provide a scientific basis for an in-depth exploration of public health prevention strategies. According to epidemiological studies, the prevalence of HHcy varied substantially with demographic characteristics, geographical distributions, and years of investigation [7,8,9]. The determinants of HHcy include genetic defects of the enzymes involved in Hcy metabolism, nutritional deficiencies of vitamin cofactors, such as folate, vitamin B12 and B6 [10], coexisting diseases [9,11], environment, and lifestyle [12].

The prevalence of HHcy in China has been determined and was presented in a previously published meta-analysis [13]. In summary, the authors concluded that the overall pooled prevalence of HHcy among the Chinese population from 1990 to 2012 was 27.5% [95% confidence interval (95% CI): 23.3–31.6%], which is relatively higher when compared with contemporaneously developed countries such as Switzerland [14] and Korea [15]. Studies investigated during later years (2006–2012) presented a higher prevalence of HHcy. The prevalence increased with elderly populations and male subjects, and was higher in north China, inland areas, and among rural residents. In the previous meta-analysis, different diagnostic criteria for HHcy concentrations were mixed and included cutoff values of either ≥15 μmol/L or other concentrations. There were also additional limitations, as the epidemiological studies involved in exploring HHcy were mainly conducted in the north regions, with very few in the south China regions. Recently, the number of studies about Hcy levels or HHcy conducted in south China has increased substantially, which contributes to the need to re-estimate the prevalence of HHcy at the national level [16,17]. Furthermore, considering the key role of Hcy in the process of vascular injury, more studies have been conducted to prevent and treat HHcy by nutritional supplements such as folic acid or other methods [18]. However, it was revealed that the prevalence of HHcy increased over time in single studies recently published [12,19,20,21]. The authors failed to determine the contributing factors to the between-study heterogeneity in the previous meta-analysis and more studies need to be included. In view of these latest findings, it was necessary to update the meta-analysis on Hcy status in China and to understand the prevalence, trend of change over time, and its determinants.

Therefore, our study proposes to update the estimates of the prevalence of HHcy in China, to determine the trend of change, and to explore additional data to determine the potential impact factors for HHcy. The current study would provide evidence for the early prevention and reduction of HHcy in China.

## 2. Materials and Methods

### 2.1. Search Strategy

We manually searched for literature regarding population-based observational studies on the prevalence of HHcy published from January 2014 to March 2021 in China using the English databases PubMed, Embase, Web of Science, and the Chinese CNKI (Chinese National Knowledge Infrastructure), CBM (Chinese Biomedical Literature Database), VIP, and Wanfang electronic databases. The keywords used for the search were (“homocysteine” OR “homocysteinemia” OR “hyperhomocysteinemia”) AND (“China” OR “Chinese”) AND (“prevalence” OR “incidence”) AND (“epidemiology” OR “cross-sectional study” OR “survey”). To find additional relevant studies, the reference lists of the identified studies were also checked thoroughly.

### 2.2. Study Selection and Inclusion and Exclusion Criteria

**Figure 2:**
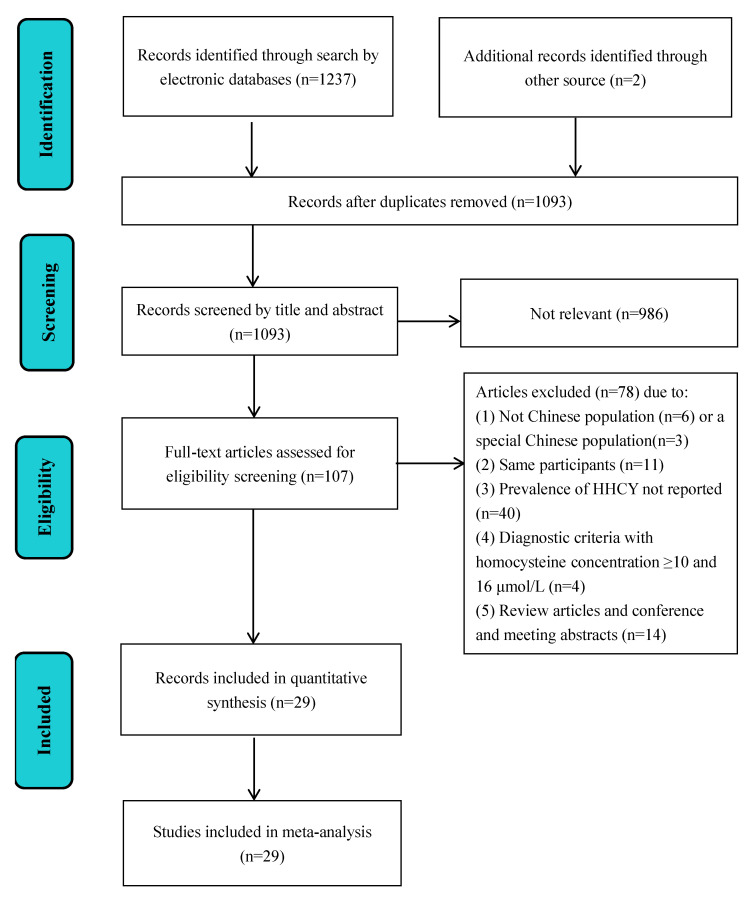
PRISMA flow diagram outlining literature search, inclusion, and exclusion of the studies.

Two reviewers independently completed identifying, screening, and including studies, as shown in Figure 2. Any dispute in these processes was discussed and determined with a third reviewer. Inclusion criteria were: (1) HHcy prevalence investigated within China and the Chinese population; (2) cross-sectional study, or baseline cross-sectional data from a cohort or an experimental trial study; (3) if there were several articles studying the same participants, the one with more detailed data would be selected; (4) data with the total number of participants and number of cases of HHcy or prevalence of HHcy reported; (5) diagnostic criteria were Hcy concentration ≥ or >15 μmol/L [1]. Exclusion criteria were: (1) data related to special populations; (2) review papers or conference and meeting abstracts; (3) HHcy prevalence not clearly reported or duplicated; (4) irrelevant or focuses on other diseases; (5) low evaluation score (risk of bias assessment), i.e., ≤3 points [22,23].

### 2.3. Date Extraction and Risk of Bias Assessment

Two reviewers independently completed data extraction and the risk of bias evaluation, and any difference in these processes was discussed and determined with a third reviewer. The extracted information was: name of the first author, year of publication, year of data collection (if the data were collected in a period, the end of this period was taken as the year of data collection, for example, if the data was collected from September 2013 to June 2014, the data collection year was recorded as 2014), mean age, population source (community based and physical examination based), sample size, number of HHcy cases, prevalence of HHcy (including prevalence in males and females, respectively), region or province (north, central or south), area (inland or coast), and setting (rural, urban, rural and urban).

In this meta-analysis, the potential bias of the included studies was assessed by using the Agency for Healthcare Research and Quality (AHRQ) [22]. The bias risk assessment consisted of a list of 11 items, including investigation sources, inclusion and exclusion criteria, time periods, continuous subjects, objectivity of indicators, repeatability of outcome indicators, reasons for exclusion of study subjects, control of confounding factors, missing data processing, response rate and data collection integrity, and follow-up results. “Yes” is given 1 point, “No” or “Unclear” is given 0 points. A total score of ≥8 is considered high quality, 4–7 is considered medium quality, and ≤3 is considered low quality [23].

### 2.4. Statistical Analysis

The statistical analysis was performed using a Stata software package version 11.0 program (Stata Corp LP, College Station, TX, USA) and SPSS Statistics version 19.0 for Windows (SPSS Inc., Chicago, IL, USA). All statistical analysis carried out in this study used the two-tailed method, and *p* values less than 0.05 were considered statistically significant, unless otherwise stated. The I^2^ test was used to assess heterogeneity [24]. According to the results of heterogeneity, the random effects model was chosen to estimate the pooled prevalence of HHcy and corresponding 95% CI by an approximate normal distribution method [25]. Subgroup analysis was conducted by demographic characteristics [age (<55 and ≥55 years) and gender (male and female)], geographical distributions [region (northern, central, and southern), area (inland and coastal), and setting (rural, urban, rural and urban)], and study characteristics [sample size (<2000 and ≥2000), year of data collection (2002–2012, 2013–2014, 2015–2016, and 2017–2018), publication (1990–2005 and 2006–2012), population source (community based and physical examination based), and research topic (main outcome and secondary outcome)]. We also used chi-square tests [26,27] to analyze subgroup differences by mean age, sex, region, area, setting, sample size, year of data collection, year of publication, population source, and research topic, and *p* for comparison <0.05 was considered statistically significant. In meta-regression analysis, independent variables included mean age, sex ratio (male to female sample size ratio), study area (northern, central, and southern), study location (inland and coastal), study setting (rural, urban, rural and urban), sample size, data collection year, publication year, population source (community based, physical examination based), and research topic (main outcome and secondary outcome). Among these variables, mean age, sex ratio (male/female), sample size, data collection year, and publication year are continuous variables. First, we performed univariate meta-regression analysis for all variables. Second, we selected those variables with *p* < 0.1 in univariate meta-regression analysis for further multivariate analysis to explore the source of heterogeneity [28]. Egg’s test was used to assess publication bias [29] and sensitivity analysis to assess the stability and robustness of the study.

## 3. Results

The current meta-analysis finally comprised 29 studies (9 from English and 20 from Chinese databases), involving 338,660 participants aged 20–74 years, among whom 128,147 had positive cases of HHcy. Prevalence data collected from 2002–2018, covering 13 provinces and 3 municipalities of China (in total 23 provinces, 4 municipalities, 5 autonomous regions, and 2 special administrative regions). A basic description of these studies is provided in Supplementary Table S1. The scores of the risk of bias evaluation in the included studies were 5–9 points, as shown in Supplementary Table S2. Of the included studies, 13 were of high quality and 16 were of medium quality.

### 3.1. Pooled Prevalence of HHcy and Subgroup Analysis

**Figure 3:**
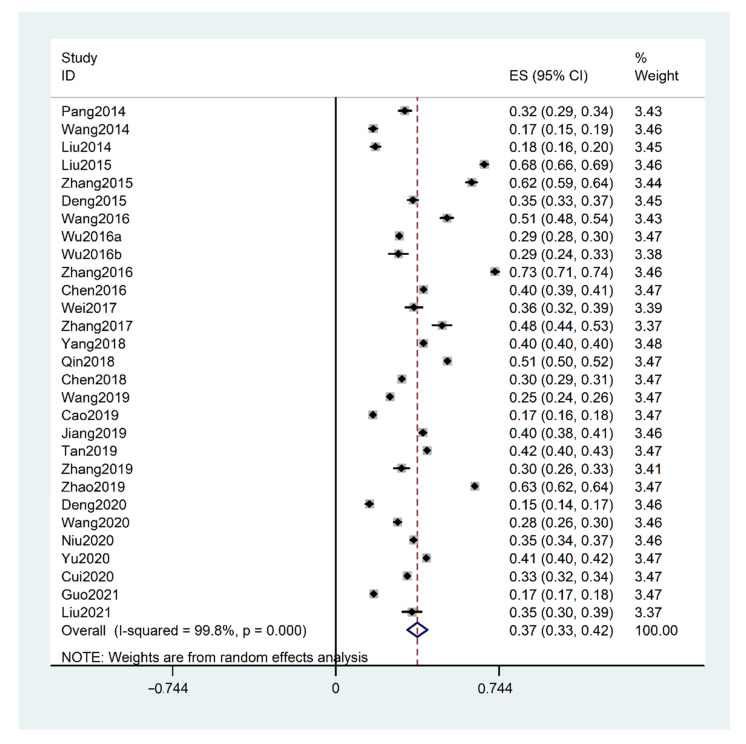
Forest plot of the prevalence of hyperhomocysteinemia (HHcy) in China. (Note: Wu, 2016a presents Wu, Q.Q., et al. Sichuan Med. J. 2016, 37, 783–787 and Wu, 2016b represents Wu, Y.Z., et al. Acad. J. Second Military Med. Univ. 2016, 37, 936–942).

The pooled prevalence of HHcy in China was 37.2% (95% CI: 32.6–41.8%, I^2^ = 99.8%, *p* for heterogeneity < 0.001), as shown in Figure 3. According to subgroup analysis as shown in Table 1, the prevalence of HHcy was higher in the elderly (age ≥ 55 vs. <55 years, *p* for comparison < 0.001), males (male vs. female, *p* for comparison < 0.001), north China (north vs. central or south China, *p* for comparison < 0.001; central vs. southern, *p* for comparison < 0.001), inland area (inland vs. coast, *p* for comparison < 0.001), rural (rural vs. urban, *p* for comparison < 0.001), sample size ≥ 2000 (sample size < 2000 vs. ≥2000, *p* for comparison < 0.001), community based (community based vs. physical examination based, *p* for comparison < 0.001), and HHcy is the secondary outcome (main outcome vs. secondary outcome, *p* for comparison < 0.001).

According to the trend of changes in the prevalence of HHcy in China over time, years of data collection of the included studies were synthesized and divided into four groups (2002–2012, 2013–2014, 2015–2016, and 2017–2018). As the current meta-analysis was a follow-up to a previous study (1990–2012), the years of 2013–2018 were a particular focus. The results showed that the trend of prevalence of HHcy increased over time and increased during 2015–2016 (*p* for comparison to 2013–2014 < 0.001) but remained steady between 2015–2015 and 2017–2018.

### 3.2. Meta-Regression Analyses to Explore the Source of Heterogeneity

To explore the potential source of heterogeneity, meta-regression analyses were performed. We found that the influencing factors of heterogeneity included age, region of north China, and coast area with *p* < 0.1 with univariate meta-regression analysis (Table 2). Further multivariate meta-regression analysis indicated that age and area of study where data were collected could explain 42.3% of the heterogeneity (Table 3).

### 3.3. Publication Bias and Sensitivity Analysis

We investigated the existence of publication bias using the Egger’s test and a funnel plot. Results of both methods (Egger’s test: *p* = 0.773 > 0.001, funnel plot: Supplementary Figure S1) indicated no publication bias. The results of sensitivity analysis also showed that the results were relatively stable (Supplementary Figure S2).

## 4. Discussion

The current meta-analysis mainly demonstrated that the estimate of the prevalence of HHcy was 37.2% in China, with a gradual increase from 2013 to 2018. The positive rate of HHcy among Chinese populations varied with age and gender, higher among the elderly and men. HHcy was more prevalent in the north region, inland area, and within rural residents of China. The current study provides evidence that HHcy is still prevalent and has kept increasing in China during the last 20 years. Prevention strategies, such as folic acid supplements to lower Hcy, were necessary to control HHcy and related public health issues.

The prevalence of HHcy in China was updated to 37.2% in our study, as compared with the previous rate of 27.5% prior to 2012, as reported by Yang et al. [13]. It remains high when compared with other developed countries, such as the United States (6.9%) [30] and Canada (19.1%) [31], but lower than Iran (73.1%) [32] and Africa (62.3%) [33]. Both subgroup analyses on the year of data collection and publication showed that the prevalence of HHcy varied substantially over time. Subsequently, time-trend analysis according to the year of data collection demonstrated that from 2013 to 2018, the prevalence of HHcy showed an upward trend from 30.9% to 35.8% and was steady at 34.1%.

Referring to the related determinants of HHcy, we achieved consistent conclusions as previous studies, with higher prevalence in the elderly over 55 years old (41.2%) and men (53.0%). Age was proven to affect the prevalence of HHcy in the study by Xu et al., which found that the concentration of Hcy in people aged 30–50 years was the lowest, increasing over the age of 50 [34]. The elderly may suffer from declined liver and kidney function due to insulted digestion and absorption in the process of Hcy metabolism [34]. In addition, high prevalence of HHcy among men might be due to more muscle mass, insufficient intake of fruits and vegetables resulting in low folic acid and vitamin content, and unhealthy lifestyle habits such as smoking, alcohol consumption, and lack of sleep [7,35,36,37].

Another interesting finding was that the geographical distribution difference between the studies also affected the prevalence of HHcy among Chinese populations. HHcy was more common in north China (45.7%), inland area (41.6%), and rural residents (42.3%), and is consistent with other studies [17,37]. The geographic difference of HHcy prevalence in different areas of China could be related to genetic defects of the enzymes involved in Hcy metabolism [8,38], nutritional deficiencies of vitamin cofactors such as folic acid [39], vitamin B12 and B6 [40], coexistence diseases [41], environment, and lifestyle [42,43] in different areas. Methylenetetrahydrofolate reductase (MTHFR) is an important enzyme reducing Hcy metabolism, which leads to HHcy [8,41]. The MTHFR 677 genotype, related to the serum concentration of Hcy [44], is characterized by the 677T allele and higher frequencies were found in north China [45]. Nutritional supplementation was another important factor. Residents in south China usually had higher intakes of folic acid and vitamin B [40,46]. In south China, especially the coastal area, there are abundant aquatic and seafood products rich in betaine and folic acid [38,39]. This result indicates that folic acid needs to be supplemented in the north and inland areas of China. The current subgroup analysis showed that the prevalence of HHcy was higher in rural residents, which was in contrast to Hao’s study [40]. With the rapid economic development and industrialization in China, dietary habits and lifestyles changed among rural and urban residents. As the current meta-analysis was limited to 13 provinces and 3 municipalities of China, more large-scale representative epidemiological national survey data should be conducted in the future to verify whether there were differences in the prevalence of HHcy between rural and urban areas of China. Other factors, such as the coexistence of hypertension, smoking, and physical activity levels, might also be responsible for the geographic differences in the prevalence of HHcy, and further experimental studies are essential [19].

Accurate estimate and temporal evolution of the prevalence of HHcy was crucial for the planning, monitoring, and management of public health interventions on HHcy-related health problems. On the basis of ensuring consistency of results with the previous meta-analysis, the strengths of this study were: (1) to update the Hcy status in recent years, and we observed a slightly increasing trend of HHcy prevalence in China; (2) to include more studies investigating the prevalence of HHcy in south China; (3) to perform meta-regression analysis on age and area (inland/coast) to determine the source, which could explain 42.3% of the between-study heterogeneity. There were some limitations in our study. According to Figure 2, 95% CI in individual studies was narrow, and the heterogeneity of the prevalence in different studies is very large. The reason for the narrow 95% CI may be related to the fact that only one set of data was considered and the sample size was large [24]. However, high heterogeneity is a limitation of single-rate meta-analysis, different studies represented different populations, and the populations included in the studies are of different age stages. Therefore, subgroup analysis and meta-regression were used to explore the source of the heterogeneity. There was also large bias in the provinces selected to investigate HHcy in China. Hcy status has never been studied in some provinces such as Xizang, Guizhou, and Yunnan [13,47]. The prevalence of HHcy reported in other studies could be quite different due to different sampling methods, population inclusion criteria, study location, social economic status, and preventive policies established in each province. Therefore, there might be a gap between the pooled prevalence of HHcy and the true prevalence in the real world, which indicates that we should be cautious about interpreting or applying the findings of the present study.

Additionally, the sample size of some included studies varied (ranging from 438 to 207,069), and studies with low samples were less representative. Therefore, future studies are needed to better understand HHcy in the following areas: (1) to develop a set of unified HHcy diagnostic criteria suitable for China, which could accurately reflect the prevalence of HHcy in China; (2) all regions should pay attention to the prevalence of HHcy and perform a high-quality epidemiological investigation, providing reliable scientific evidence for the prevention, treatment, and control of HHcy in China.

## 5. Conclusions

The current meta-analysis provided strong evidence that the prevalence of HHcy has increased in China, especially among the elderly, men, residents in the north, inland areas, and rural parts of China. Therefore, high-risk populations should be the focus of public health policies, and preventive strategies should be performed and implemented to prevent and control the increase in HHcy in China.

## Figures and Tables

**Table 1:** Pooled prevalence of HHcy in China by different stratified factors.

Subgroups	Sample Size	No. of Studies	Pooled Prevalence (%) (95% CI)	Heterogeneity		*p* for Comparison
				I^2^	*p*	
**Demographic characteristics**						
**Mean age (year)**		24				<0.001
<55	247,175	13	34.5 (29.1–40.0)	99.7%	<0.001	
≥55	63,447	11	41.2 (30.6–51.8)	99.9%	<0.001	
**Gender**						<0.001
male	165,303	20	53.0 (45.6–60.4)	99.8%	<0.001	
female	135,585	20	28.0 (22.6–33.4)	99.8%	<0.001	
**Geographical distributions**						
**Region**		29				<0.001
north	34,017	11	45.7 (35.7–55.7)	99.8%	<0.001	
central	275,702	12	32.3 (27.3–37.4)	99.8%	<0.001	
south	28,941	6	31.1 (16.3–45.9)	99.8%	<0.001	
**Area**		29				<0.001
inland	50,584	14	41.6 (31.3–51.8)	99.8%	<0.001	
coast	288,076	15	33.1 (27.5–38.6)	99.8%	<0.001	
**Setting**		29				<0.001
rural	23,667	7	42.3 (33.1–51.5)	99.5%	<0.001	
urban	47,453	8	33.1 (25.6–40.5)	99.7%	<0.001	
urban and rural	267,540	14	36.9 (30.1–43.7)	99.9%	<0.001	
**Study characteristics**						
**Sample size**		29				<0.001
<2000	12,370	12	33.9 (24.8–42.9)	99.3%	<0.001	
≥2000	326,290	17	39.5 (33.6–45.4)	99.9%	<0.001	
**Year of data collection**		25				<0.001
2002–2012	16,055	6	49.2 (36.9–61.6)	99.6%	<0.001	
2013–2014	50,412	9	30.9 (24.5–37.3)	99.6%	<0.001	
2015–2016	245,640	6	35.8 (27.5–44.2)	99.9%	<0.001	
2017–2018	20,033	4	34.1 (14.2–55.5)	99.8%	<0.001	
**Year of publication**		29				<0.001
2014–2017	33,569	13	41.4 (31.4–51.1)	99.7%	<0.001	
2018–2021	305,091	16	33.9 (27.9–39.8)	99.9%	<0.001	
**Population source**		29				<0.001
Community based	82,874	16	39.4 (32.4–46.5)	99.8%	<0.001	
physical examination based	255,786	13	34.4 (27.4–41.3)	99.8%	<0.001	
**Research topic**		29				<0.001
Main outcome (HHcy)	85,109	23	37.1 (30.7–43.5)	99.8%	<0.001	
Secondary outcome (HHcy)	253,551	6	37.4 (28.1–46.6)	99.9%	<0.001	

HHcy, hyperhomocysteinemia, and diagnostic criteria with homocysteine concentration ≥ or >15 μmol/L; 95% CI, 95% confidence intervals.

**Table 2:** Univariate meta-regression analysis for prevalence of HHcy in China.

Variables	Sample Size	No. of Studies	Coefficient	Lower 95% CI	Upper 95% CI	*p*
**Demographic characteristics**						
Mean age	300,424	18	0.01	0.004	0.02	0.039
Male/female	300,424	18	−0.04	−0.25	0.17	0.683
**Geographical distributions**						
**Region**	300,424	18				
south	18,712	3	Reference			
north	27,026	7	0.24	0.05	0.42	0.016
central	254,686	8	0.05	−0.13	0.23	0.539
**Area**	300,424	18				
inland	32,399	8	Reference			
coast	268,025	10	−0.15	−0.29	−0.01	0.034
**Setting**	300,424	18				
rural	17,560	4	Reference			
urban	34,426	4	−0.07	−0.31	0.17	0.545
rural and urban	248,438	10	−0.10	−0.30	0.10	0.303
**Study characteristics**						
Sample size	300,424	18	−8.26 × 10^−8^	−1.08 × 10^−6^	1.64 × 10^−6^	0.920
Year of data collection	300,424	18	−0.01	−0.03	0.01	0.313
Year of publication	300,424	18	−0.03	−0.06	0.01	0.101
**Population source**	300,424	18				
physical examination based	236,089	8	Reference			
Community based	64,335	10	0.02	−0.18	0.14	0.754
**Research topic**	300,424	18				
Secondary outcome (HHcy)	231,205	3	Reference			
Main outcome (HHcy)	69,219	15	0.06	−0.27	0.15	0.537

HHcy, hyperhomocysteinemia, and diagnostic criteria with homocysteine concentration ≥ or >15 μmol/L; 95% CI, 95% confidence intervals.

**Table 3:** Multivariate meta-regression analysis for prevalence of HHcy in China.

Variables	Sample Size	No. of Studies	Coefficient	Lower 95% CI	Upper 95% CI	*p*	R-Squared
Mean age	300,424	18	0.008	0.002	0.01	0.018	42.3%
Area	300,424	18					
inland	32,399	8	Reference				
coast	268,025	10	−0.15	−0.27	−0.03	0.017	

HHcy, hyperhomocysteinemia, and diagnostic criteria with homocysteine concentration ≥ or >15 μmol/L; 95% CI, 95% confidence intervals.

## Data Availability

The authors confirm that the data supporting the findings of this work are available within the article.

## Supplementary Materials

The following are available online at https://www.mdpi.com/article/10.3390/biology10100959/s1, Figure S1: Funnel chart, Figure S2: Sensitivity analyses, Table S1: Basic characteristics of studies on the prevalence of HHcy in China, Table S2: Risk of bias assessment of selected studies.
