# Supplementary material for: Prevalence of Hyperhomocysteinemia in China: An Updated Meta-Analysis
Source: Biology (Basel). 2021 Sep 26;10(10):959. doi: 10.3390/biology10100959 (PMC8533293; doi:10.3390/biology10100959)
Supplement: Supplementary file 1 [file biology-10-00959-s001.zip › biology-1323731-supplementary.pdf]

Supplementary Table S1. Basic characteristics of studies on the prevalence of HHcy (defined as Hcy ≥ 15μmol/L) in China

Authors	Year of Publication	year of data collection	Population source	Age (Years)	Province	Setting	Area	Study Location	Methods of Hcy measurement	Sample	Number of Cases	Prevalence of HHCY (%)	Prevalence of male (%)	Prevalence of female (%)
Pang2014[1]	2014	2011.5-2012.11	Community-based	42.2±11.4	Xinjiang	R	N	I	ECA	1003	316	31.5	49.9	17.5
Wang2014[2]	2014	NR	Community-based	54.05±12.11	Guangdong	U	S	Co	ECA	1586	270	17.02	29.6	8.47
Liu2014[3]	2014	2012.08-2013.09	Physical examination -based	43. 04 ± 10. 24	Jilin	U and R	N	I	ECA	1106	200	18.1	NR	NR
Liu2015[4]	2015	2007	Community-based	43.57	Shanxi	U and R	N	I	ECA	2645	1791	67.7	81.4	58.8
Zhang2015[5]	2015	2009	Community-based	69.5±8.1	Beijing	U	N	I	ECA	1458	902	61.87	NR	NR
Deng2015[6]	2015	NR	Community-based	72.96± 7.66	Shanghai	R	C	Co	HPLC	1621	569	35.1	43.6	29.5
Wang2016[7]	2016	2013.1-2013.12	Physical examination -based	49.1±11.7	Guangdong	U and R	S	Co	IA	1136	575	50.61	65.61	27.78
Wu2016a[8]	2016	2013.1-2014.10	Physical examination -based	45. 86 ±13. 53	Sichuan	U and R	C	I	ECA	11740	3393	28.9	40.53	8.77
Wu2016b[9]	2016	2014.1	Physical examination -based	38.0±8.6	Shanghai	U and R	C	Co	ECA	438	125	28.54	NR	NR
Zhang2016[10]	2016	2012.8-2012.12	Community-based	64.6±7.4	Shanxi	R	N	I	ECA	2355	1710	72.6	84.3	63.2
Chen2017[11]	2017	2012.7-2013.8	Community-based	53.8±10.6	Liaoning	R	N	I	ECA	7365	2944	39.97	59.04	25.79
Zhan2017[12]	2017	2012.8-2012.12	Community-based	63.68	Shanxi	U	N	I	ECA	516	250	48.4	NR	NR
Wei2017[13]	2017	2015-2016	Community-based	≥35	Guangxi	R	S	Co	ECA	600	214	35.7	40.4	28.1

Yang2018[14]	2018	2013-2018	Physical examination -based	54.8±7.9	Hunan	U and R	C	Co	NR	207069	82701	39.94	55.6	16.7
Qin2018[15]	2018	2015-2016	Physical examination -based	≥20	Guangxi	U and R	S	Co	ECA	8043	4086	50.8	52.3	48.1
Chen2018[16]	2020	2016.08-2016.09	Community-based	61.2±9.5	Jiangsu	U	C	Co	ECA	16644	5018	30.15	43.65	22.31
Wang2019[17]	2019	2013	Community-based	55.7 ± 11.1	Shanghai	U and R	C	Co	ECA	8337	2059	24.7	NR	NR
Cao2019[18]	2019	2015.07-2017.12	Physical examination -based	NR	Chongqing	U and R	C	I	ECA	7308	1237	16.93	90.54	5.6
Jiang2019[19]	2019	2007	Community-based	35-64	Jiangxi	R	C	I	ECA	3886	1542	39.68	49.61	24.14
Tan2019[20]	2019	2015	Physical examination -based	72.57±6.51	Shanghai	U and R	C	Co	ECA	5976	2487	41.62	51.47	33.82
Zhang2019[21]	2019	NR	Physical examination -based	61.91±7.26	Heilongjiang	U	N	I	ELISA	668	200	29.94	25.99	33.72
Zhao2019[22]	2019	2017.01	Physical examination -based	59.8±9.8	Xinjiang	U and R	N	I	NR	7492	4745	63.33	74.23	54.46
Deng2020[23]	2020	2017.07-2018.10	Physical examination -based	50.37±6.09	Guangxi	U and R	S	Co	ECA	1768	270	15.2	23.1	6
Wang2020[24]	2020	2018.01-2018.06	Physical examination -based	58.15±11.74	Hebei	U	N	I	NR	2572	724	28.15	NR	NR
Yu2020[25]	2020	2001.01-2003.08	Community-based	54 ± 10	Liaoning	R	N	Co	ECA	6837	2824	41.3	NR	NR
Cui2020[26]	2020	2016.06-2017.11	Community-based	20-74	Shanghai	U	C	Co	ECA	8201	2673	32.59	52.69	18.6
Niu2021[27]	2021	2013-2014	Community-based	54.5	Hunan	U and R	C	Co	NR	4012	1420	35.39	45.38	28.46

Guo2021[28]	2021	2012.03-2015.07	Community-based	57.5±10.9	Guangdong	U	S	Co	NR	15808	2739	17.33	NR	NR
Liu2021[29]	2021	2014.04-2015.04	Physical examination -based	40±8.4	Sichuan	U and R	C	I	ELISA	470	163	34.68	NR	NR

HHcy, hyperhomocysteinemia; Hcy, homocysteine; NR, not reported; N, north; S, south; Ce, central; I, inland; Co, coastal; U, urban; R, rural; HPLC, high-performance liquid chromatography; ECA, enzymatic cycling assay; IA, immunoturbidimetric assays.

Table S2. Quality scores for assessing the risk of bias of in the included articles for observation study.

Study(year)	Q1	Q2	Q3	Q4	Q5	Q6	Q7	Q8	Q9	Q10	Q11	Total Score
Pang2014[1]	1	0	1	1	1	0	1	1	0	1	1	8
Wang2014[2]	1	1	1	1	1	0	1	0	0	1	1	8
Liu2014[3]	1	0	1	1	1	0	0	0	0	0	1	5
Liu2015[4]	1	1	1	1	1	0	1	1	0	1	1	9
Zhang2015[5]	1	1	1	1	1	0	1	1	0	1	1	9
Deng2015[6]	1	0	0	1	1	0	0	0	0	1	1	5
Wang2016[7]	1	1	1	1	1	0	0	0	0	0	1	6
Wu2016a[8]	1	0	1	1	1	0	0	0	0	0	1	5
Wu2016b[9]	1	1	1	1	1	0	1	0	0	1	1	8
Zhang2016[10]	1	1	1	1	1	0	1	0	0	1	1	8
Chen2017[11]	1	1	1	1	1	0	0	1	0	1	1	8
Zhan2017[12]	1	1	1	1	1	0	1	1	0	1	1	9
Wei2017[13]	1	1	1	1	1	0	0	0	0	0	1	6
Yang2018[14]	1	1	1	1	1	0	0	0	0	0	1	6
Qin2018[15]	1	1	1	1	1	0	0	1	0	0	1	7

Chen2018[16]	1	1	1	1	1	0	0	0	0	0	1	6
Wang2019[17]	1	1	1	1	1	0	0	1	0	0	1	7
Cao2019[18]	1	1	1	1	1	0	0	1	0	0	1	7
Jiang2019[19]	1	0	1	1	1	0	0	0	0	0	1	5
Tan2019[20]	1	0	1	1	1	0	1	0	0	1	1	7
Zhang2019[21]	1	1	0	1	1	0	1	0	0	1	1	7
Zhao2019[22]	1	1	1	1	1	0	0	0	0	0	1	6
Deng2020[23]	1	1	1	1	1	0	0	1	0	0	1	7
Wang2020[24]	1	1	1	1	1	0	1	0	0	1	1	8
Yu2020[25]	1	1	1	1	1	0	1	1	0	1	1	9
Cui2020[26]	1	1	1	1	1	0	1	1	0	1	1	9
Niu2020[27]	1	1	1	1	1	0	1	1	0	1	1	9
Guo2021[28]	1	1	1	1	1	0	0	1	0	1	1	8
Liu2021[29]	1	1	1	1	1	0	0	0	0	0	1	6

"No" or "Unclear" was scored "0", and "Yes" was scored "1". Low quality: ≤3 scores; Moderate quality: 4-7 scores; High quality: ≥8 scores. Q1: Define the source of information (survey, record review); Q2: List inclusion and exclusion criteria for exposed and unexposed subjects (cases and controls) or refer to previous publications; Q3: Indicate time period used for identifying patients; Q4: Indicate whether or not subjects were consecutive if not population-based; Q5: Indicate if evaluators of subjective components of study were masked to other aspects of status of the participants; Q6: Describe any assessments undertaken for quality assurance purpose; Q7: Explain any patient exclusions from analysis; Q8: Describe how confounding was assessed and/or controlled; Q9: If applicable, explain how missing data were handled in the analysis; Q10: Summarize patient response rates and completeness of data collection; Q11: Clarify what follow-up, if any, was expected and the percentage of patients for which incomplete data or follow-up was obtained.

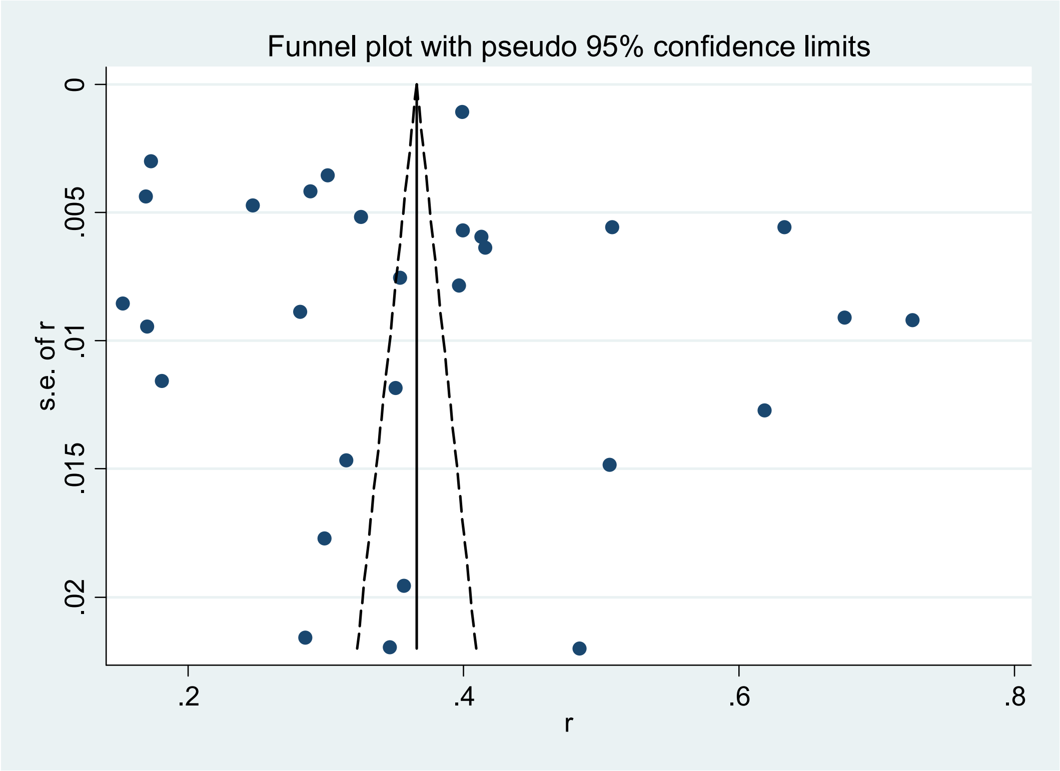

Supplementary Figure S1. Funnel chart

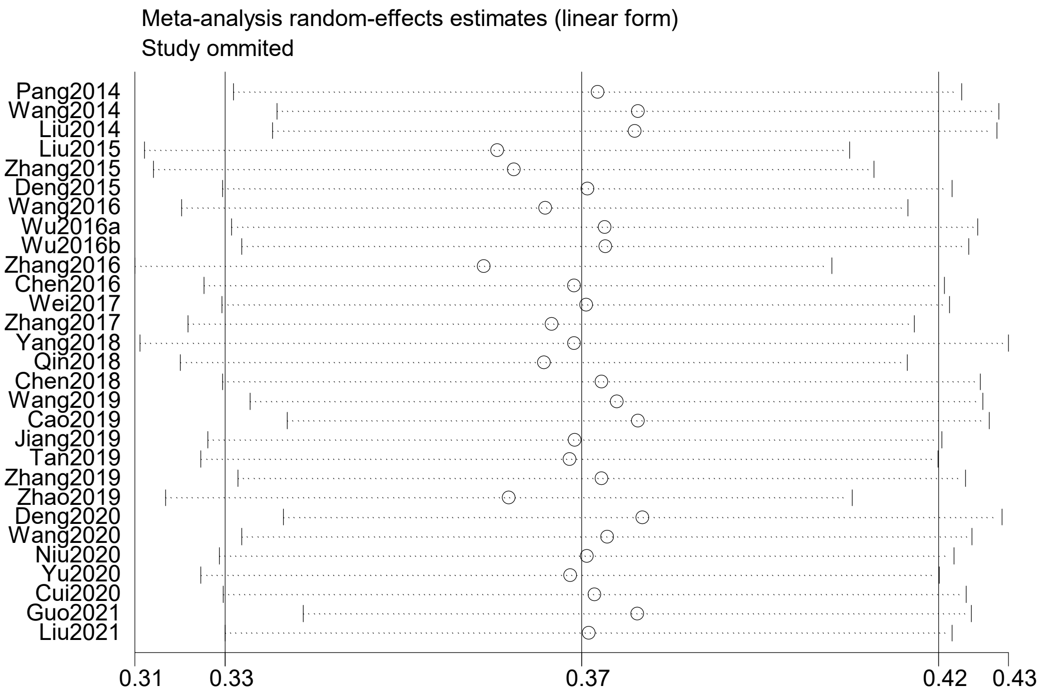

Supplementary Figure S2. Sensitivity analyses

1. Pang,R.H.; Zhang,J.Y.; Xu,S.Z.; Ma,R.L.; Guo,H.; Liu,J.M.; Zhang,M.; Ding,Y.S.; Li,S.G.; Guo,S,X. The Epidemiological Characteristics of High Serum Level of Homocysteine in Kazakh Population at Xinyuan County of Xinjiang. Chin Circ J. 2014, 29, 913-916.
2. Wang,C.Y.; Zhang,T.; Chen,S.H.; Ma,J.P.; Liu,S.Y.; Peng,X.L.; Peng,J.; Xu,J.; Chen,Z.W. Study on the distribution of hyperhomocysteinemia and its influencing factors in healthy population of community in Nanshan Shenzhen. J Trop Med. 2014, 14, 1204-1208.
3. Liu, .R.; Jiang, W.X.; Song, C.X.; Zheng, W.M.; Tan L. Hyperhomocysteinemia and its associated risk factors in healthy subjects. Chinese journal of clinical research. 2014. 27. 637-638.
4. Liu, X.D.; Gao, B.; Sun, D.; Shi, M.;Ma, Y.Y.; Liu, Z.R.; Wang, B.; Xu, X.; Xu, X.; Ji, Q.H.; Zhao, G. Prevalence of hyperhomocysteinaemia and some of its major determinants in Shaanxi Province, China: a cross-sectional study. Br J Nutr. 2015, 113, 691-698. http://doi.org/10.1017/s0007114514004218
5. Zhang,Z.Y.;Fang,X.H.;Jie,X.H.;Tang,Z.;Guan,S.C.;Liu,H.J.;Wu,X.G.;Wang,C.X.;Hou,C.B.;Gu,X.The association between serum homocysteine and blood pressure among middle-aged and elderly people. Chin J hypertens. 2015, 23, 846-850.
6. Deng,Q.Q.;Gao,L.L.;Fu,J.H.; Pan,D.H.; Xiao,W.Z.; Li,Q.H.; Ding,D.; Pan,X.J.; Yu,B. The prevalence and risk factors of hyperhomocysteinemia in elderly people in rural community of Shanghai. Fudan Univ J Med Sci. 2015, 42, 722-726.
7. Wang,J.F.; Ma,X.T.; Liu,G.Z.; Xie,T.; Xie,Y.L. The characteristic of total plasma total homocysteine level distribution in population of Zhaihai area. Chongqing Med J. 2016, 45, 3972-3974.xx
8. Wu,Q.Q.; Tang,H.R.; Tang,G.H.; Li,Q.; Xue,G.C.; Chen,Y.; Leng,R.;Wang,Y.J. The Cross-sectional Survey of the Serum Homocysteine in Chengdu Area. Sichuan Med J. 2016, 37, 783-787.
9. Wu,Y,Z; Lu,D.R.; Chen,X.X.; Jiang,Y.M. Correlation of homocysteine levels with gene polymorphisms of metabolic enzyme in 438 adults taking physical examination in Shanghai,china.Acad J Second Military Med Univ. 2016, 37, 936-942.
10. Zhang, J.;Liu, T.T.;Zhang, W.;Li, Y.;Niu, X.Y.;Fang, Y.L.;Ma, L.S.;Li, C.X. Hyperhomocysteinemia Is Associated with Vitamin B-12 Deficiency: A Cross-sectional Study in a Rural, Elderly Population of Shanxi China. J Nutr Health Aging. 2016, 20, 594-601. http://doi.org/10.1007/s12603-015-0650-511
11. Chen, S.;Guo, X.;Dong, S.;Li, Z.;Sun, Y. Relationship between lifestyle factors and hyperhomocysteinemia in general Chinese population: a cross-sectional study. Postgrad Med. 2017, 129, 216-223. http://doi.org/10.1080/00325481.2017.1255126
12. Zhang, H.; Shang, S.H.; Deng, M.Y.; Yan, F.; Li, Z.L.;, Chen, C.; Jiang, Y.; Dang, L.J.; Zhu, J.; Li, Y.; Qu, Q.M. Correlation between plasma homocysteine level and blood pressure in the high-risk population of stroke in the community of Xi'an. Chinese Journal of Integrative Medicine on Cardio-Cerebrovascular Disease. 2017,15, 3186-3193.

13. Wei,S.W.; Wei,Y.L.; Xun,W.H.; Wei,X.Y. Prevalence and Influencing Factors of Hyperhomocysteinemia in Baiku Yao Nationality Residents in Guangxi. PJCCPVD. 2017, 25, 73-75.
14. Yang,B.F. Correlation analysis between overweight and obesity and plasma homocysteine and blood lipid levels in healthy people. Chin commun doc. 2018, 34, 122-123.
15. Qin, Y.Y.;Wang, P.;Qin, J.Q.;Wei, A.Q.;Huang, P.;Lai, Z.F.;Lin, F.Q. Prevalence of hyperhomocysteinemia during routine physical examination in Guangxi Province, China and related risk factors. J Clin Lab Anal. 2018, 32. http://doi.org/10.1002/jcla.22178
16. Chen, L.;Wang, B.;Wang, J.;Ban, Q.;Wu, H.;Song, Y.;Zhang, J.;Cao, J.;Zhou, Z.;Liu, L.;Cao, T.;Gao, L.;Guo, H.;Zhang, T.;Tang, G.;Huang, X.;Zhang, Y.;Li, J.;Huo, Y.;Cheng, X.;Zang, T.;Xu, X.;Zhang, H.;Qin, X. Association between serum total homocysteine and arterial stiffness in adults: a community-based study. J Clin Hypertens (Greenwich). 2018, 20, 686-693. http://doi.org/10.1111/jch.13246
17. Wang, Y.H.;Yan, Q.H.;Xu, J.Y.;Li, X.J.;Cheng, M.N. High Prevalence and Factors Contributing to Hyperhomocysteinemia, Folate Deficiency, and Vitamin B12 Deficiency among Healthy Adults in Shanghai, China. Biomed Environ Sci. 2019, 32, 63-67. http://doi.org/10.3967/bes2019.010
18. Cao,Y.;Li,B.;He,J.T.; Yue,Y.; Wang,X.H.; Chen,Y.; Jia,S.Y. The relationship between serum homocysteine level and changes of cerebral hemodynamic indexes. Chongqing Med J. 2019, 48, 1225-1227+1230.
19. Jiang,L.X.; Dong,M.H.; Shi,S.H.; Zhang,H.J.; Li,S.M.; Xia,Y; Cao,J. The study of environmental factors for hyperhomocysteinemia in the population of Ganzhou. J Gannan Med Univ. 2019, 39, 762-765.
20. Tan,Q.; Wang,J.G.; Li,Y.; Wang,D.; Chen,Y.; Guo,H.Q. Clinical Characteristics of Hyperhomocysteinemia at Various Diagnostic Cut-off Thresholds. Prevention and Treatment of Cardio-Cerebral-Vascular Disease. 2019, 19, 292-295.
21. Zhang,Y.; Ying,X.;Guo,Y.p.; Chen,Y.L.; Wang,J.P.; Wang,Z.G. Analysis of influencing factors of homocysteine in middle-aged and elderly population. J Harbin Med univ. 2019, 53, 598-601.
22. Zhao,Y.; Shang,L.X.; Zhang,W.H.; Li,Y.D.; Zhou,X.H.;Tang,B.P. Correlation between metabolic syndrome components and hyperhomocysteinemia in residents with physical examination of Xinjiang area. Chin J Prevent Control Chronic Dis. 2019, 27, 425-427+431.
23. Deng,Z.K.; Ming,Y.; Li,H.B.; Lin,H.L.; Yang,J.; Bo,F. Analysis on the relationship between hyperhomocysteinemia and blood lipid and BMI in healthy people taking medical examination in Guilin . Chinese Journal of Healthcare and Medicine. 2020, 22, 599-602.
24. Wang,Z.L.; Zhang,X.M.; Hao,Z.H.; Nie,Q.; Liu,C.H.; Liu,X.X.;Wang,L.L. Relationship between serum homocysteine and metabolic syndrome in middle-aged and aged people of state-run institutions. Pract Geriatr. 2020, 34, 1165-1169.
25. Yu, S.;Chen, Y.;Yang, H.;Guo, X.;Zheng, L.;Sun, Y. Hyperhomocysteinemia accompany with metabolic syndrome increase the risk of left ventricular hypertrophy in

rural Chinese. BMC Cardiovasc Disord. 2020, 20, 44. http://doi.org/10.1186/s12872-020-01350-2
26. Cui,S.H.; Zhao,Q.; Wang,N.; Zhang,Y.; Qiu,Y.; Zhou,X.Y.; Yu,Y.T.; Wang,R.P.; Jiang,Y.G.; Zhao,G.M. Association between metabolic syndrome and its components with hyperhomocysteinemia in community population. Chin J Dis Control & Prev. 2020, 24, 1003-1008.
27. Niu, X.;Chen, J.;Wang, J.;Li, J.;Zeng, D.;Wang, S.;Hong, X. A Cross-sectional Study on the Relationship Between Homocysteine and Lipid Profiles Among Chinese Population from Hunan. Lipids. 2021, 56, 93-100. http://doi.org/10.1002/lipd.12279
28. Guo,Z.G.; Huang,J.Y.; Meng,R.W.; Shi,O.M.; Tie,X.H.; Mo,H.M.; Wang,Z.H. Correlation between high homocysteine and the risk of stroke in community residents aged 40 years and above in Shenzhen city. Chin J Health Manage . 2021, 15, 37-43. http://doi.org/10.3760/cma.j.cn115624-20200527-00427
29. Liu,X.Y.;Li,Y.;Liu,T. Correlation of homocysteine levels with gene polymorphisms of homocysteine metabolic enzymes in physical examination in Nan Chong. J of Pub Health and Prev Med. 2021, 32, 113-116.
